# Supplementary material for: Impact of the antidepressant citalopram on the behaviour of two different life stages of brown trout
Source: PeerJ. 2020 Mar 12;8:e8765. doi: 10.7717/peerj.8765 (PMC7073243; doi:10.7717/peerj.8765)
Supplement: Supplemental Information 1 [file peerj-08-8765-s001.docx]

# Water analysis

Table 1: Operating parameters of the triple quadrupole MS (Agilent 6490 QqQ) in positive mode

| Parameter | Set point |
| --- | --- |
| Gas temperature | 250 °C |
| Gas flow | 14 L/min |
| Nebulizer gas pressure | 45 psi |
| Sheath gas heater | 300 °C |
| Sheath gas flow | 12 L/min |
| Capillary voltage | 3500 V |
| Nozzle voltage | 0 V |

Table 2: Specific measurement parameters for citalopram with LC-QqQ in water samples. Intraday variations (RSD) is calculated with 1 µg/L standard (10 µl injection volume and 4 replicates (n)). Limit of quantification= LOQ

| Precursor | Product ion (Quan/Qual) (m/z) | Collision energy (setpoint in V) | Retention time  (min) | RSD (%)  n=4 | LOQ (ng/L) |
| --- | --- | --- | --- | --- | --- |
| 325 | 262/109 | 20/30 | 3.92 | 2.6 | 10 |

# Tissue analysis

## Sample preparation

For the extraction of citalopram from fish tissue, a micro-QuEChERS (quick, easy, cheap, efficient, rugged, safe) method was applied and the extraction step was optimized by a design of experiment. Frozen fish tissue were first homogenized with mortar and pestle under liquid nitrogen. Aliquots of 25/50 mg of the homogenized sample were weighed into a 2.5 mL tube (Eppendorf, Germany) and 0.25 mL acetonitrile (Cromasolv, Merck, Darmstadt, Germany) and 0.75 mL water (Cromasolv, Merck, Darmstadt, Germany) were added. For extraction, samples were shaken with a vortex device for 30 sec., then 30 mg sodium chloride and 120 mg anhydrous magnesium sulfate (Sigma-Aldrich, Steinheim, Germany) were added and the sample immediately shaken for 30 sec. After centrifugation for 15 min at 13 000 rpm, 0.1 mL of the acetonitrile phase was evaporated to dryness under a gentle stream of nitrogen and the concentrated residue was resolved in 0.3 mL methanol. The extracts were diluted to reach concentrations compatible with the calibration range established for citalopram. After filtration using a 45 μm PTFE filter (pore size 0.45 μm, Chromafil, Macherey-Nagel, Germany), the samples were analysed by LC–MS. Matrix-matched calibration in the range of 1-20 µg/L was used to correct for matrix effects in fish extracts. Recoveries of the extraction process were calculated by comparing the peak area of spiked fish samples and the peak area of fish samples that were spiked after the extraction process. With the optimized method, the extraction recovery of citalopram was 88 %. The limit of detection of the method was 3 ng/L, corresponding to 0.06 ng citalopram per g wet weight tissue. Intraday variations of the analytical method (*n=6*) were 1% for trout larvae matrix and 2% for juvenile brown trout matrix.

## Quantification

All analyses were performed using a 1260 Infinity LC system coupled to a 6550 iFunnel QTOF mass spectrometer (Agilent Technologies, Waldbronn, Germany and Santa Clara, CA, USA). Aliquots of 10 μL sample were injected onto a Zorbax Eclipse Plus C18 column (2.1 x 150 mm, 3.5-Micron, narrow bore, Agilent Technologies, Waldbronn, Germany) at a column temperature of 40 °C. A jet stream electrospray ionization (ESI) source was operated with a nebulizer pressure of 35 psig, drying gas temperature of 160 °C, at a flow rate of 16 L/min and a fragmentor voltage of 360 V. In the positive ionization mode capillary voltage was set to - 4000 V, skimmer voltage to 65 V and nozzle voltage to - 500 V. The mass range was 100 - 1200 m/z with a data acquisition rate of 1 spectrum/s. For internal calibration purine and HP0921 (Agilent Technologies, Waldbronn, Germany, m/z = 121.0508, 922.0097) were used. A gradient elution at a flow rate of 0.3 mL/min using water and methanol, containing 0.1 % formic acid was used. The initial content of 95 % water was decreased after 1 min to 5 % water over 7 min and after another 7 min at 5 % increased to 95 % water over 0.5 min. Data analysis was performed with MassHunter software (Agilent Technologies, Waldbronn, Germany).

# Accumulation


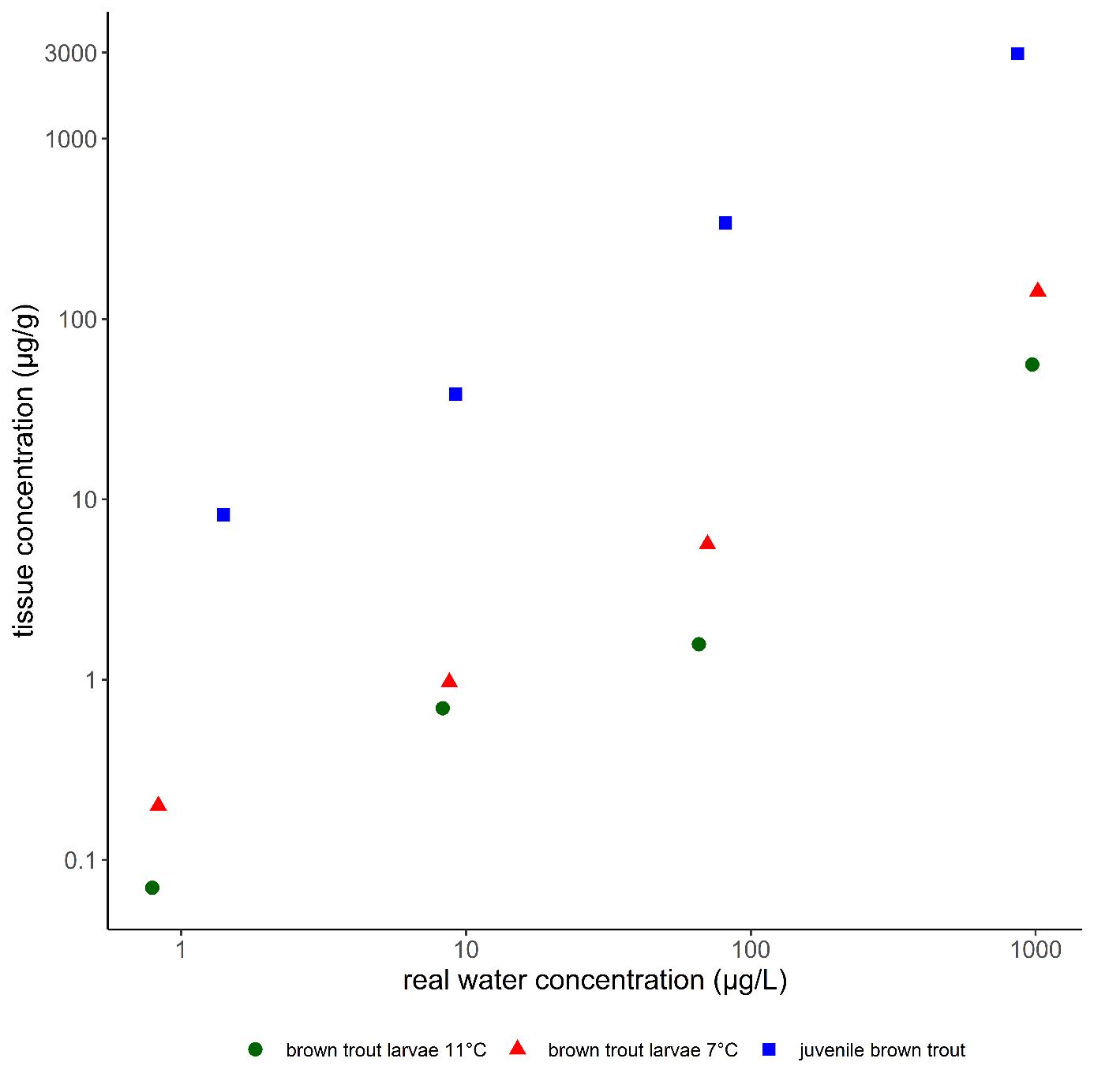


Figure 1: Citalopram water concentration plotted against citalopram tissue concentration of the expermients with brown trout larvae and juvenile brown trout. depicted are average means. ▲= brown trout larvae at 7°C, ●=brown trout larvae at 11°C, ■= juvenile brown trout at 7°C
