# Supplementary material for: Impact of the antidepressant citalopram on the behaviour of two different life stages of brown trout
Source: PeerJ. 2020 Mar 12;8:e8765. doi: 10.7717/peerj.8765 (PMC7073243; doi:10.7717/peerj.8765)
Supplement: Supplemental Information 2 [file peerj-08-8765-s002.docx]

# CRED

Table 3: Criteria for reporting and evaluating ecotoxicity data, adapted by Moermont et al. (2015)

| CRED-criteria: Exposure of brown trout larvae to citalopram | | | | | |
| --- | --- | --- | --- | --- | --- |
| 1. General information | | | | | |
| a. Purpose | | | Aim of the study was to investigate the effect of citalopram on the development and behaviour of brown trout in different life stages. | | |
| b. Endpoints | | | Time to hatch, mortality, heart rate, weight, length, Behaviour during the exposure in minor stress, behaviour in a stressful environment, biochemical determination of the tissue cortisol content, determination of the citalopram tissue concentration | | |
| 2. Test design | | | | | |
| a. Standard | | | No standard test procedure | | |
| b. GLP | | | Not GLP-accredited | | |
| c. Controls | | | Laboratory negative control | | |
| d. Validity | | | mortality in control was <10% (brown trout larvae at 7°C, juvenile brown trout)  mortality in control was <30% (brown trout larvae at 11°C) | | |
| 3. Test compound | | | | | |
| a. Identification | | Citalopram hydrobromide  **1-[3-(Dimethylamino)propyl]-1-(4-fluorophenyl)-1.3-dihydro-5-isobenzofurancarbonitrile hydrobromide**  CAS: 59729-32-7 | | |  |
| b. Physico-chemical characteristics | | water solubility of 16.56 g/L (TOCRIS 2017) | | |  |
| c. Source | | Sigma-Aldrich, Product Number: PHR1640-1G; Batch Number: LRAA6012 | | |  |
| d. Purity | | 99.8% according to the Certificate of Analysis from Sigma-Aldrich | | |  |
| e. Formulation | | no formulation, no impurities | | |  |
| 4. Test organism | | | | |  |
| a. Scientific name | | *Salmo trutta* f. *fario* | | |  |
| b. Body weight/length | | larvae  7°C: Body weight: mean=0.32 g ± 0.10  7°C: Body length: mean=3.24 cm ± 0.33  11°C: Body weight: mean=0.43 g ± 0.16  11°C: Body length: mean=3.64 cm ± 0.46  juveniles:  Body weight: mean=2.67 g ± 0.92  Body length: mean=6.38 cm ± 0.69 | | |  |
| c. Age/life stage | | From eyed ova stage until 8 weeks post yolk-sac consumption (larvae)  8 month old (juveniles) | | |  |
| d. Reproductive condition | | not in reproductive condition | | |  |
| e. Sex | | not determinable | | |  |
| f. Strain/clone | | no defined clone | | |  |
| g. Source | | commercial trout farm (Forellenzucht Lohmühle, D-72275 Alpirsbach-Ehlenbogen) | | |  |
| h. Acclimatisation | | No acclimatisation (larvae)  2 weeks in 200 l tank at 7°C (aerated filtered tap water) (juveniles) | | |  |
| 5. Exposure conditions | | | | |  |
| a. Schedule | | semi-static design with water exchange of 50 % of the test medium twice a week | | |  |
| b. System | | closed | | |  |
| c. Test medium | | Filtered tap water (iron filter, active charcoal filter, particle filter) cooled to 7°C resp. 11°C and aerated | | |  |
| d. Temperature | | Climate chamber set to 7 °C resp. 11°C.  larvae  7 °C: mean= 7.10 °C ± 0.32  11 °C: mean= 10.77 °C ± 0.30  Juveniles  mean= 7.15 °C ± 0.41 | | |  |
| e. pH | | Larvae  7 °C: mean=8.08 ± 0.41  11 °C: mean=7.96 ± 0.46  Juveniles  mean=8.09 ± 0.01 | | |  |
| f. Hardness | | not measured | | |  |
| g. Conductivity | | Larvae  7°C: mean=472.59 µS/cm ± 9.92  11°C: mean=478.28 µS/cm ± 7.21  Juveniles  mean=493.73 µs/cm ± 17.47 | | |  |
| h. Dissolved oxygen | | Larvae  7°C: mean=10.77 mg/L ± 0.30  11°C: mean=9.94 mg/L ± 0.50  juveniles  mean=11.22 mg/L ± 0.10 | | |  |
| i. Light intensity/quality | | 10 h : 14 h light:dark cycle; aquaria shaded from direct light with black foil | | |  |
| j. Feeding | | After yolk sac consumption, brown trout larvae were fed once per day with commerical trout feed (0.5 mm first 4 weeks post yolk sac consumption, then 0.8 mm (Inicio Plus, Biomar, Brande, Denmark))  Juvenile brown trout were fed once per day with commercial trout feed (8 mm, Inico Plus, Biomar, Brande, Denmark) | | |  |
| k. Aquaria | | Larvae  25 L glass aquaria filled with 10 L of medium. covered with glass plane, silicone tubing, aerated with airstones (JBL ProSilent Aeras Micro S2)  Juveniles  25 L glass aquaria filled with 15 L of medium, covered with glass plane, silicone tubing, aerated with airstones (JBL ProSilent Aeras Micro S2) | | |  |
| l. Sand/sediment | | no sediment tested | | |  |
| m. Stock solutions | | Stock solution 1 (100 mg/L) prepared from 124.94 mg citalopram hydrobromide in 1 L dest. Water, stock solution 2 (1 mg/L) produced from stock solution 1 via 1:100 dilution | | |  |
| n. Nominal concentrations | | 0. 1. 10. 100. 1000 μg/L | | |  |
| o. Measured concentration | | Larvae  Water samples were taken and analysed at the beginning, once every month before and after water exchange and at the end of the experiment.  7°C mean: < LoD, 0.83, 8.7, 70.5, 1017.9 μg/L  11°C mean: < LoD, 0.79, 8.3, 65.7, 974.0 μg/L  Juveniles  Water samples were taken and analysed at the beginning, after 2 weeks before and after water exchange and at the end of the experiment. mean: < LoD, 1.4, 9.2, 81.5, 864.9 μg/L | | |  |
| p. Method | | HPLC-MS (QqQ-MS) (LoQ = 1 ng/L) | | |  |
| q. Duration | | Larvae  7°C: 28.12.2016 – 11.05.2017  11°C: 28.12.2016 – 13.04.2017  Juveniles  08.08.2017 - 4.09.2017 | | |  |
| r. Observations | | Mortality, hatching rate, heart rate, behaviour during the exposure under minor stress, behaviour in a stressful environment. body length and body weight, at the end of the experiment samples were taken for biochemical and analytical analyses | | |  |
| s. Results | | summary table in article | | |  |
| t. Biomass loading | | Larvae  7°C: mean= 0.96 g/L  11°C: mean=1.29 g/L  Juveniles  mean: 1.78 g/L | | |  |
| 6. Statistical Design and Biological Response | | | |  |  |
| a. Replicates | Three replicate aquaria per test concentration and temperature | | |  |  |
| b. Number of organisms | 30 fish per replicate , 10 fish per replicate | | |  |  |
| c. Design | In each climate chamber: three blocks, one replicate per treatment present in each block, arranged in randomized order | | |  |  |
| d. Statistical methods | Mortality and hatching rate: nested Cox proportional hazards model; body length/mass, heart rate, behaviour in stressful environment: nested ANOVA; behaviour during exposure: Generalized Liner Mixed Model; Cortisol content: Linear Mixed Model | | |  |  |
| e. Biological response | Increased body length and weight at 1000 µg/L (larvae 7+11°C and juveniles)  Increased sojourn in upper aquaria part 1000 µg/L (larvae 7°C), 1000 µg/L (larvae 11°C and juveniles)  Decreased total distance moved in stressful environment at 100, 1000 µg/L (larvae 7°C) and 1000 µg/L (larvae 11°C)  Decreased mean velocity and increased time of no movement over time at 100 and 1000 µg/L (larvae 7°C)  Trend to decreased mean velocity and increased time of no movement over time (larvae 11°C)  70% swimming activity in stressful environment in all treatments compared to control (juveniles)  Increased tissue cortisol content in fish measured in the artificial swimming measurement device (juveniles) | | |  |  |
| f. Dose-response | Decreased swimming activity in stressful environment 100 and 1000 µg/L (larvae 7°C) | | |  |  |
| g. Statistical significances | Increased body length and weight at 1000 µg/L (larvae 7+11°C and juveniles)  Increased sojourn in upper aquaria part 1,10,1000 µg/L (larvae 7°C), 1000 µg/L (larvae 11°C and juveniles  Decreased total distance moved in stressful environment at 100, 1000 µg/L (larvae 7°C) and 1000 µg/L (larvae 11°C)  Decreased mean velocity and increased time of no movement over time at 100 and 1000 µg/L (larvae 7°C)  Trend to decreased mean velocity and increased time of no movement over time (larvae 11°C) | | |  |  |
| h. Significance level | α = 0.05, in cases of multiple comparisons adjusted via sequential Bonferroni | | |  |  |
| i. Variability | not estimated | | |  |  |
| j. Raw data | provided on request | | |  |  |
