# Supplementary material for: Impact of the antidepressant citalopram on the behaviour of two different life stages of brown trout
Source: PeerJ. 2020 Mar 12;8:e8765. doi: 10.7717/peerj.8765 (PMC7073243; doi:10.7717/peerj.8765)
Supplement: Supplemental Information 3 [file peerj-08-8765-s003.docx]

# Statistical details

Table 9: detailed statistical information to the respective parameter

| parameter | experiment | | detailed statistical information | |
| --- | --- | --- | --- | --- |
| weight | brown trout larvae 7 °C | | square root transformation: nested ANOVA: df=4.10; F=9.7415; p<0.0001; post-hoc Dunnett's Test [control\|1000 µg/L] p<0.0001 | |
| weight | brown trout larvae 11 °C | | fourth root transformation: nested ANOVA: df=4.10; F=12.8137; p<0.0001; post-hoc Dunnett's Test [control\|1000µg/L] p<0.0001 | |
| weight | juvenile brown trout | | logarithm transformation: nested ANOVA: df=4.10; F=3.2964; p=0.0013; post-hoc Dunnett's Test [control\|1000µg/L] p=0.0234 | |
| length | brown trout larvae 7 °C | | nested ANOVA: df=4.10; F=22.0216; p<0.0001; post-hoc Dunnett's Test [control\|1000µg/L] p<0.0001 | |
| length | brown trout larvae 11 °C | | nested ANOVA: df=4.10; F=13.1786; p<0.0001; post-hoc Dunnett's Test [control\|1000µg/L] p<0.0001 | |
| lenght | juvenile brown trout | | nested ANOVA: df=4.10; F=4.6661; p=0.0015; post-hoc Dunnett's Test [control\|1000µg/L] p=0.0193 | |
| heart rate | brown trout larvae 7 °C | | nested ANOVA: df=4.9; F=2.6161; p=0.0447; post-hoc Dunnett's Test no difference to control | |
| heart rate | brown trout larvae 11 °C | | nested ANOVA: df=1.4; F=0.3968; p=0.5347 | |
| mortality | brown trout larvae 7 °C | | nested Cox Regression: df=4.10; χ2=6.9203; p=0.1402 | |
| mortality | brown trout larvae 11 °C | | nested Cox Regression: df=4.10; χ2=4.2743; p=0.3701 | |
| time to hatch | brown trout larvae 7 °C | | nested Cox Regression: df=4.10; χ2=2.42E-0.9; p=1 | |
| time to hatch | brown trout larvae 11 °C | | nested Cox Regression: df=4.10; χ2=0; p=1 | |
| sojourn in upper aquaria half | brown trout larvae 7 °C | | Generalized Linear Mixed Model, df=4, F= 106.9664, post-hoc Dunnett's [0 µg/L\|1µg/L] p=0,0265, [0 µg/L\|10 µg/L] p=0.0165, [0 µg/L\|1000 µg/L] p<0.001 | |
| sojourn in upper aquaria half | brown trout larvae 11 °C | | Generalized Linear Mixed Model, df=4, F= 12.4141, post-hoc Dunnett's [0 µg/L\|1000 µg/L] p=0,000000865 | |
| sojourn in upper aquaria half | juvenile brown trout | | Generalized linear mixed model, df=4, F= 7.3259, post-hoc Dunnett's [0 µg/L\|1000 µg/L] p=0,00107 | |
| ASMD: total distance moved | brown trout larvae 7 °C | | square root transformation: nested ANOVA: df=4.10; F=7.8214; p<0.0001; post-hoc Dunnett's Test [control\|100µg/L] p=0.0212; [control\|1000µg/L] p<0.0001 | |
| ASMD: total distance moved | brown trout larvae 11 °C | | nested ANOVA: df=4.10; F=4.7551; p=0.0021; post-hoc Dunnett's Test [control\|1000µg/L] p=0.0008 | |
| ASMD: total distance moved | juvenile brown trout | | logarithm transformation: nested ANOVA: df=4.10; F=1.0846; p=0.3818 | |
| ASMD: mean velocity over time | brown trout larvae 7 °C | | Linear mixed model: df=4;10, F=5.6897, p=0.01186; post-hoc Dunnett’s Test [control\|100 µg/L] p=0.0236 [control\|1000 µg/L] p<0.001 | |
| ASMD: mean velocity over time | brown trout larvae 11 °C | | Linear mixed model: df=4;10, F=2.7222, p=0.09068 | |
| ASMD: mean velocity over time | juvenile brown trout | | Linear mixed model: df=4;10, F=0.4324, p=0.7824 | |
| ASMD:  Time of no movement | brown trout larvae 7 °C | | Linear mixed model: df=4;10, F=5.4797, p=0.01338; post-hoc Dunnett’s Test [control\|100 µg/L] p=0.0414 [control\|1000 µg/L] p<0.001 | |
| ASMD:  Time of no movement | brown trout larvae 11 °C | | Linear mixed model: df=4;10, F=3.1072, p=0.06643 | |
| ASMD:  Time of no movement | juvenile brown trout | | Linear mixed model: df=4;10, F=0.4902, p=0.7433 | |
| Cortisol content | | juvenile brown trout | | Linear Mixed Model:  difference Treatment:  df=4,68.638, F=3.7625, p=0.007959; post-hoc Dunnett’s Test no difference between control and treatments  difference Tested/not Tested:  df=1,95.378, F=16.7132, p=0.00009072 |

## Statistical output

### Behaviour during exposure brown trout larvae 7°C

Generalized linear mixed model fit by maximum likelihood (Laplace

Approximation) [glmerMod]

Family: binomial ( logit )

Formula: cbind(oben, unten) ~ Treatment + log(Tage.nach.Versuchsbeginn) +

(1 | AquariumID)

Data: Data.BFL.CIT.7

Control: glmerControl(optimizer = "bobyqa", optCtrl = list(maxfun = 1e+05))

AIC BIC logLik deviance df.resid

192.9 210.9 -89.5 178.9 89

Scaled residuals:

Min 1Q Median 3Q Max

-1.2853 -0.6436 -0.1953 0.3748 3.7213

Random effects:

Groups Name Variance Std.Dev.

AquariumID (Intercept) 0.01511 0.1229

Number of obs: 96, groups: AquariumID, 8

Fixed effects:

Estimate Std. Error z value Pr(>|z|)

(Intercept) -0.2165 3.9113 -0.055 0.95585

Treatment1 2.6982 1.0824 2.493 0.01267 *

Treatment10 2.7749 1.0420 2.663 0.00775 **

Treatment100 2.1395 1.1308 1.892 0.05850 .

Treatment1000 7.6848 1.0155 7.567 3.81e-14 ***

log(Tage.nach.Versuchsbeginn) -1.3030 0.8128 -1.603 0.10893

---

Signif. codes: 0 ‘***’ 0.001 ‘**’ 0.01 ‘*’ 0.05 ‘.’ 0.1 ‘ ’ 1

Correlation of Fixed Effects:

(Intr) Trtmn1 Trtm10 Trt100 Tr1000

Treatment1 -0.237

Treatment10 -0.246 0.895

Treatmnt100 -0.228 0.825 0.857

Tretmnt1000 -0.233 0.919 0.954 0.879

lg(Tg.nc.V) -0.966 -0.001 -0.002 0.000 -0.022

Analysis of Variance Table

Df Sum Sq Mean Sq F value

Treatment 4 427.87 106.966 106.9664

log(Tage.nach.Versuchsbeginn) 1 2.57 2.567 2.5671

Simultaneous Tests for General Linear Hypotheses

Multiple Comparisons of Means: Dunnett Contrasts

Fit: glmer(formula = cbind(oben, unten) ~ Treatment + log(Tage.nach.Versuchsbeginn) +

(1 | AquariumID), data = Data.BFL.CIT.7, family = binomial,

control = glmerControl(optimizer = "bobyqa", optCtrl = list(maxfun = 1e+05)))

Linear Hypotheses:

Estimate Std. Error z value Pr(>|z|)

1 - 0 == 0 2.698 1.082 2.493 0.0265 *

10 - 0 == 0 2.775 1.042 2.663 0.0165 *

100 - 0 == 0 2.139 1.131 1.892 0.1101

1000 - 0 == 0 7.685 1.016 7.567 <0.001 ***

---

Signif. codes: 0 ‘***’ 0.001 ‘**’ 0.01 ‘*’ 0.05 ‘.’ 0.1 ‘ ’ 1

(Adjusted p values reported -- single-step method)

### Behaviour during exposure brown trout larvae 11°C

Generalized linear mixed model fit by maximum likelihood (Laplace

Approximation) [glmerMod]

Family: binomial ( logit )

Formula: cbind(oben, unten) ~ Treatment + log(Tage.nach.Versuchsbeginn) +

(1 | AquariumID)

Data: Data.BFL.CIT.11

Control: glmerControl(optimizer = "bobyqa", optCtrl = list(maxfun = 1e+05))

AIC BIC logLik deviance df.resid

190.7 203.7 -89.4 178.7 58

Scaled residuals:

Min 1Q Median 3Q Max

-2.8812 -0.6775 -0.4835 0.4889 3.3617

Random effects:

Groups Name Variance Std.Dev.

AquariumID (Intercept) 0.3666 0.6055

Number of obs: 64, groups: AquariumID, 8

Fixed effects:

Estimate Std. Error z value Pr(>|z|)

(Intercept) 25.2462 15.9740 1.580 0.1140

Treatment10 -0.1648 0.7696 -0.214 0.8305

Treatment100 0.4254 0.7478 0.569 0.5694

Treatment1000 3.4542 0.7021 4.920 8.65e-07 ***

log(Tage.nach.Versuchsbeginn) -6.0778 3.4691 -1.752 0.0798 .

---

Signif. codes: 0 ‘***’ 0.001 ‘**’ 0.01 ‘*’ 0.05 ‘.’ 0.1 ‘ ’ 1

Correlation of Fixed Effects:

(Intr) Trtm10 Trt100 Tr1000

Treatment10 -0.024

Treatmnt100 -0.023 0.483

Tretmnt1000 0.009 0.523 0.520

lg(Tg.nc.V) -0.999 0.001 0.001 -0.034

Analysis of Variance Table

Df Sum Sq Mean Sq F value

Treatment 3 37.242 12.4141 12.4141

log(Tage.nach.Versuchsbeginn) 1 3.042 3.0418 3.0418

Simultaneous Tests for General Linear Hypotheses

Multiple Comparisons of Means: Dunnett Contrasts

Fit: glmer(formula = cbind(oben, unten) ~ Treatment + log(Tage.nach.Versuchsbeginn) +

(1 | AquariumID), data = Data.BFL.CIT.11, family = binomial,

control = glmerControl(optimizer = "bobyqa", optCtrl = list(maxfun = 1e+05)))

Linear Hypotheses:

Estimate Std. Error z value Pr(>|z|)

10 - 0 == 0 -0.1648 0.7696 -0.214 0.993

100 - 0 == 0 0.4254 0.7478 0.569 0.894

1000 - 0 == 0 3.4542 0.7021 4.920 <1e-05 ***

---

Signif. codes: 0 ‘***’ 0.001 ‘**’ 0.01 ‘*’ 0.05 ‘.’ 0.1 ‘ ’ 1

(Adjusted p values reported -- single-step method)

### Behaviour during exposure juvenile brown trout

Generalized linear mixed model fit by maximum likelihood (Laplace

Approximation) [glmerMod]

Family: binomial ( logit )

Formula: cbind(oben, unten) ~ Treatment + log(Tage.nach.Versuchsbeginn) +

(1 | AquariumID)

Data: Data.JBF.CIT

AIC BIC logLik deviance df.resid

118.2 137.0 -52.1 104.2 101

Scaled residuals:

Min 1Q Median 3Q Max

-1.4591 -0.2321 -0.1845 -0.1418 3.4665

Random effects:

Groups Name Variance Std.Dev.

AquariumID (Intercept) 0.4648 0.6818

Number of obs: 108, groups: AquariumID, 9

Fixed effects:

Estimate Std. Error z value Pr(>|z|)

(Intercept) -1.063e+01 2.384e+00 -4.459 8.24e-06 ***

Treatment1 1.957e-04 1.595e+00 0.000 0.999902

Treatment10 7.345e-01 1.671e+00 0.439 0.660328

Treatment100 -6.437e-06 1.595e+00 0.000 0.999997

Treatment1000 4.483e+00 1.244e+00 3.604 0.000314 ***

log(Tage.nach.Versuchsbeginn) 1.653e+00 6.889e-01 2.399 0.016437 *

---

Signif. codes: 0 ‘***’ 0.001 ‘**’ 0.01 ‘*’ 0.05 ‘.’ 0.1 ‘ ’ 1

Correlation of Fixed Effects:

(Intr) Trtmn1 Trtm10 Trt100 Tr1000

Treatment1 -0.334

Treatment10 -0.322 0.477

Treatmnt100 -0.334 0.500 0.477

Tretmnt1000 -0.457 0.641 0.615 0.641

lg(Tg.nc.V) -0.879 0.000 0.001 0.000 0.025

Analysis of Variance Table

Df Sum Sq Mean Sq F value

Treatment 4 29.3035 7.3259 7.3259

log(Tage.nach.Versuchsbeginn) 1 5.7325 5.7325 5.7325

Simultaneous Tests for General Linear Hypotheses

Multiple Comparisons of Means: Dunnett Contrasts

Fit: glmer(formula = cbind(oben, unten) ~ Treatment + log(Tage.nach.Versuchsbeginn) +

(1 | AquariumID), data = Data.JBF.CIT, family = binomial)

Linear Hypotheses:

Estimate Std. Error z value Pr(>|z|)

1 - 0 == 0 1.957e-04 1.595e+00 0.000 1.00000

10 - 0 == 0 7.345e-01 1.671e+00 0.439 0.97365

100 - 0 == 0 -6.437e-06 1.595e+00 0.000 1.00000

1000 - 0 == 0 4.483e+00 1.244e+00 3.604 0.00107 **

---

Signif. codes: 0 ‘***’ 0.001 ‘**’ 0.01 ‘*’ 0.05 ‘.’ 0.1 ‘ ’ 1

(Adjusted p values reported -- single-step method)

### Mean velocity over time brown trout larvae 7°C

Linear mixed model fit by REML. t-tests use Satterthwaite's method ['lmerModLmerTest']

Formula: mean.velocity.cm.s ~ treatment + time + (1 | Aq_ID)

Data: BFL.CIT.7.2_20

REML criterion at convergence: 4428.8

Scaled residuals:

Min 1Q Median 3Q Max

-2.0741 -0.7282 -0.1731 0.5866 3.8406

Random effects:

Groups Name Variance Std.Dev.

Aq_ID (Intercept) 0.1994 0.4466

Residual 1.5063 1.2273

Number of obs: 1350, groups: Aq_ID, 15

Fixed effects:

Estimate Std. Error df t value Pr(>|t|)

(Intercept) 1.361e+00 2.753e-01 1.107e+01 4.944 0.000432 ***

treatment1 -8.174e-01 3.796e-01 1.000e+01 -2.153 0.056751 .

treatment10 -6.313e-01 3.796e-01 1.000e+01 -1.663 0.127272

treatment100 -1.030e+00 3.796e-01 1.000e+01 -2.713 0.021831 *

treatment1000 -1.764e+00 3.796e-01 1.000e+01 -4.648 0.000911 ***

time 9.777e-02 6.438e-03 1.334e+03 15.185 < 2e-16 ***

---

Signif. codes: 0 ‘***’ 0.001 ‘**’ 0.01 ‘*’ 0.05 ‘.’ 0.1 ‘ ’ 1

Correlation of Fixed Effects:

(Intr) trtmn1 trtm10 trt100 tr1000

treatment1 -0.689

treatment10 -0.689 0.500

treatmnt100 -0.689 0.500 0.500

tretmnt1000 -0.689 0.500 0.500 0.500

time -0.222 0.000 0.000 0.000 0.000

Type III Analysis of Variance Table with Satterthwaite's method

Sum Sq Mean Sq NumDF DenDF F value Pr(>F)

treatment 34.28 8.57 4 10 5.6897 0.01186 *

time 347.32 347.32 1 1334 230.5747 < 2e-16 ***

---

Signif. codes: 0 ‘***’ 0.001 ‘**’ 0.01 ‘*’ 0.05 ‘.’ 0.1 ‘ ’ 1

Simultaneous Tests for General Linear Hypotheses

Multiple Comparisons of Means: Dunnett Contrasts

Fit: lmer(formula = mean.velocity.cm.s ~ treatment + time + (1 | Aq_ID),

data = BFL.CIT.7.2_20)

Linear Hypotheses:

Estimate Std. Error z value Pr(>|z|)

1 - 0 == 0 -0.8174 0.3796 -2.153 0.1015

10 - 0 == 0 -0.6313 0.3796 -1.663 0.2783

100 - 0 == 0 -1.0298 0.3796 -2.713 0.0236 *

1000 - 0 == 0 -1.7644 0.3796 -4.648 <0.001 ***

---

Signif. codes: 0 ‘***’ 0.001 ‘**’ 0.01 ‘*’ 0.05 ‘.’ 0.1 ‘ ’ 1

(Adjusted p values reported -- single-step method)

Groups Name Variance

Aq_ID (Intercept) 0.19943

Residual 1.50631

### Time of no movement over time brown trout larvae 7°C

Linear mixed model fit by REML. t-tests use Satterthwaite's method ['lmerModLmerTest']

Formula: cummulative.no.movement.s ~ treatment + time + (1 | Aq_ID)

Data: BFL.CIT.7.2_20

REML criterion at convergence: 11216.4

Scaled residuals:

Min 1Q Median 3Q Max

-2.8834 -0.7052 0.1381 0.7632 2.1985

Random effects:

Groups Name Variance Std.Dev.

Aq_ID (Intercept) 36.9 6.074

Residual 234.8 15.324

Number of obs: 1350, groups: Aq_ID, 15

Fixed effects:

Estimate Std. Error df t value Pr(>|t|)

(Intercept) 42.94910 3.70842 10.90508 11.581 1.82e-07 ***

treatment1 11.62578 5.13210 9.99994 2.265 0.04694 *

treatment10 9.01519 5.13210 9.99994 1.757 0.10950

treatment100 12.88981 5.13210 9.99994 2.512 0.03083 *

treatment1000 23.67770 5.13210 9.99994 4.614 0.00096 ***

time -1.29498 0.08039 1334.00002 -16.109 < 2e-16 ***

---

Signif. codes: 0 ‘***’ 0.001 ‘**’ 0.01 ‘*’ 0.05 ‘.’ 0.1 ‘ ’ 1

Correlation of Fixed Effects:

(Intr) trtmn1 trtm10 trt100 tr1000

treatment1 -0.692

treatment10 -0.692 0.500

treatmnt100 -0.692 0.500 0.500

tretmnt1000 -0.692 0.500 0.500 0.500

time -0.206 0.000 0.000 0.000 0.000

Type III Analysis of Variance Table with Satterthwaite's method

Sum Sq Mean Sq NumDF DenDF F value Pr(>F)

treatment 5147 1287 4 10 5.4797 0.01338 *

time 60937 60937 1 1334 259.5111 < 2e-16 ***

---

Signif. codes: 0 ‘***’ 0.001 ‘**’ 0.01 ‘*’ 0.05 ‘.’ 0.1 ‘ ’ 1

Simultaneous Tests for General Linear Hypotheses

Multiple Comparisons of Means: Dunnett Contrasts

Fit: lmer(formula = cummulative.no.movement.s ~ treatment + time +

(1 | Aq_ID), data = BFL.CIT.7.2_20)

Linear Hypotheses:

Estimate Std. Error z value Pr(>|z|)

1 - 0 == 0 11.626 5.132 2.265 0.0780 .

10 - 0 == 0 9.015 5.132 1.757 0.2342

100 - 0 == 0 12.890 5.132 2.512 0.0414 *

1000 - 0 == 0 23.678 5.132 4.614 <0.001 ***

---

Signif. codes: 0 ‘***’ 0.001 ‘**’ 0.01 ‘*’ 0.05 ‘.’ 0.1 ‘ ’ 1

(Adjusted p values reported -- single-step method)

Groups Name Variance

Aq_ID (Intercept) 36.899

Residual 234.814

### Mean velocity over time brown trout larvae 11°C

Linear mixed model fit by REML. t-tests use Satterthwaite's method ['lmerModLmerTest']

Formula: mean.velocity.cm.s ~ treatment + time + (1 | Aq_ID)

Data: BFL.CIT.11.2_20

REML criterion at convergence: 4409.6

Scaled residuals:

Min 1Q Median 3Q Max

-2.1637 -0.7490 -0.1109 0.7066 2.9449

Random effects:

Groups Name Variance Std.Dev.

Aq_ID (Intercept) 0.3357 0.5794

Residual 1.4793 1.2163

Number of obs: 1350, groups: Aq_ID, 15

Fixed effects:

Estimate Std. Error df t value Pr(>|t|)

(Intercept) 2.013e+00 3.479e-01 1.064e+01 5.785 0.000139 ***

treatment1 -6.598e-01 4.845e-01 1.000e+01 -1.362 0.203137

treatment10 -1.837e-01 4.845e-01 1.000e+01 -0.379 0.712558

treatment100 -4.144e-01 4.845e-01 1.000e+01 -0.855 0.412401

treatment1000 -1.451e+00 4.845e-01 1.000e+01 -2.994 0.013477 *

time 5.126e-02 6.381e-03 1.334e+03 8.034 2.06e-15 ***

---

Signif. codes: 0 ‘***’ 0.001 ‘**’ 0.01 ‘*’ 0.05 ‘.’ 0.1 ‘ ’ 1

Correlation of Fixed Effects:

(Intr) trtmn1 trtm10 trt100 tr1000

treatment1 -0.696

treatment10 -0.696 0.500

treatmnt100 -0.696 0.500 0.500

tretmnt1000 -0.696 0.500 0.500 0.500

time -0.174 0.000 0.000 0.000 0.000

Type III Analysis of Variance Table with Satterthwaite's method

Sum Sq Mean Sq NumDF DenDF F value Pr(>F)

treatment 16.108 4.027 4 10 2.7222 0.09068 .

time 95.474 95.474 1 1334 64.5383 2.065e-15 ***

---

Signif. codes: 0 ‘***’ 0.001 ‘**’ 0.01 ‘*’ 0.05 ‘.’ 0.1 ‘ ’ 1

Simultaneous Tests for General Linear Hypotheses

Multiple Comparisons of Means: Dunnett Contrasts

Fit: lmer(formula = mean.velocity.cm.s ~ treatment + time + (1 | Aq_ID),

data = BFL.CIT.11.2_20)

Linear Hypotheses:

Estimate Std. Error z value Pr(>|z|)

1 - 0 == 0 -0.6598 0.4845 -1.362 0.45504

10 - 0 == 0 -0.1837 0.4845 -0.379 0.98711

100 - 0 == 0 -0.4144 0.4845 -0.855 0.80702

1000 - 0 == 0 -1.4507 0.4845 -2.994 0.00992 **

---

Signif. codes: 0 ‘***’ 0.001 ‘**’ 0.01 ‘*’ 0.05 ‘.’ 0.1 ‘ ’ 1

(Adjusted p values reported -- single-step method)

Groups Name Variance

Aq_ID (Intercept) 0.33568

Residual 1.47935

### Time of no movement over time brown trout larvae 11°C

Linear mixed model fit by REML. t-tests use Satterthwaite's method ['lmerModLmerTest']

Formula: cummulative.no.movement.s ~ treatment + time + (1 | Aq_ID)

Data: BFL.CIT.11.2_20

REML criterion at convergence: 11229.3

Scaled residuals:

Min 1Q Median 3Q Max

-2.86521 -0.76047 0.05118 0.74184 2.37499

Random effects:

Groups Name Variance Std.Dev.

Aq_ID (Intercept) 62.16 7.884

Residual 236.23 15.370

Number of obs: 1350, groups: Aq_ID, 15

Fixed effects:

Estimate Std. Error df t value Pr(>|t|)

(Intercept) 34.90086 4.70975 10.55069 7.410 1.7e-05 ***

treatment1 8.90095 6.57191 10.00000 1.354 0.2054

treatment10 0.88915 6.57191 10.00000 0.135 0.8951

treatment100 5.70576 6.57191 10.00000 0.868 0.4056

treatment1000 20.30020 6.57191 10.00000 3.089 0.0115 *

time -0.67370 0.08063 1334.00000 -8.356 < 2e-16 ***

---

Signif. codes: 0 ‘***’ 0.001 ‘**’ 0.01 ‘*’ 0.05 ‘.’ 0.1 ‘ ’ 1

Correlation of Fixed Effects:

(Intr) trtmn1 trtm10 trt100 tr1000

treatment1 -0.698

treatment10 -0.698 0.500

treatmnt100 -0.698 0.500 0.500

tretmnt1000 -0.698 0.500 0.500 0.500

time -0.163 0.000 0.000 0.000 0.000

Type III Analysis of Variance Table with Satterthwaite's method

Sum Sq Mean Sq NumDF DenDF F value Pr(>F)

treatment 2936 734 4 10 3.1072 0.06643 .

time 16492 16492 1 1334 69.8161 < 2e-16 ***

---

Signif. codes: 0 ‘***’ 0.001 ‘**’ 0.01 ‘*’ 0.05 ‘.’ 0.1 ‘ ’ 1

Simultaneous Tests for General Linear Hypotheses

Multiple Comparisons of Means: Dunnett Contrasts

Fit: lmer(formula = cummulative.no.movement.s ~ treatment + time +

(1 | Aq_ID), data = BFL.CIT.11.2_20)

Linear Hypotheses:

Estimate Std. Error z value Pr(>|z|)

1 - 0 == 0 8.9010 6.5719 1.354 0.46000

10 - 0 == 0 0.8892 6.5719 0.135 0.99976

100 - 0 == 0 5.7058 6.5719 0.868 0.79895

1000 - 0 == 0 20.3002 6.5719 3.089 0.00739 **

---

Signif. codes: 0 ‘***’ 0.001 ‘**’ 0.01 ‘*’ 0.05 ‘.’ 0.1 ‘ ’ 1

(Adjusted p values reported -- single-step method)

Groups Name Variance

Aq_ID (Intercept) 36.899

Residual 234.814

### Mean velocity over time juvenile brown trout larvae

Linear mixed model fit by REML. t-tests use Satterthwaite's method ['lmerModLmerTest']

Formula: mean.velocity.cm.s ~ treatment + time + (1 | Aq_ID)

Data: JBF.CIT.2_20

REML criterion at convergence: 2325.7

Scaled residuals:

Min 1Q Median 3Q Max

-2.2007 -0.6361 -0.1906 0.3203 4.1068

Random effects:

Groups Name Variance Std.Dev.

Aq_ID (Intercept) 0.2497 0.4997

Residual 0.9775 0.9887

Number of obs: 810, groups: Aq_ID, 15

Fixed effects:

Estimate Std. Error df t value Pr(>|t|)

(Intercept) 0.521647 0.305497 10.926640 1.708 0.116

treatment1 -0.485463 0.422568 9.999975 -1.149 0.277

treatment10 -0.456447 0.422568 9.999975 -1.080 0.305

treatment100 -0.400312 0.422568 9.999975 -0.947 0.366

treatment1000 -0.361975 0.422568 9.999975 -0.857 0.412

time 0.078744 0.006696 794.000009 11.760 <2e-16 ***

---

Signif. codes: 0 ‘***’ 0.001 ‘**’ 0.01 ‘*’ 0.05 ‘.’ 0.1 ‘ ’ 1

Correlation of Fixed Effects:

(Intr) trtmn1 trtm10 trt100 tr1000

treatment1 -0.692

treatment10 -0.692 0.500

treatmnt100 -0.692 0.500 0.500

tretmnt1000 -0.692 0.500 0.500 0.500

time -0.208 0.000 0.000 0.000 0.000

Type III Analysis of Variance Table with Satterthwaite's method

Sum Sq Mean Sq NumDF DenDF F value Pr(>F)

treatment 1.691 0.423 4 10 0.4324 0.7824

time 135.190 135.190 1 794 138.3017 <2e-16 ***

---

Signif. codes: 0 ‘***’ 0.001 ‘**’ 0.01 ‘*’ 0.05 ‘.’ 0.1 ‘ ’ 1

Simultaneous Tests for General Linear Hypotheses

Multiple Comparisons of Means: Dunnett Contrasts

Fit: lmer(formula = mean.velocity.cm.s ~ treatment + time + (1 | Aq_ID),

data = JBF.CIT.2_20)

Linear Hypotheses:

Estimate Std. Error z value Pr(>|z|)

1 - 0 == 0 -0.4855 0.4226 -1.149 0.604

10 - 0 == 0 -0.4564 0.4226 -1.080 0.654

100 - 0 == 0 -0.4003 0.4226 -0.947 0.747

1000 - 0 == 0 -0.3620 0.4226 -0.857 0.806

(Adjusted p values reported -- single-step method)

Groups Name Variance

Aq_ID (Intercept) 0.24974

Residual 0.97750

### Time of no movement over time juvenile brown trout larvae

Linear mixed model fit by REML. t-tests use Satterthwaite's method ['lmerModLmerTest']

Formula: cummulative.no.movement.s ~ treatment + time + (1 | Aq_ID)

Data: JBF.CIT.2_20

REML criterion at convergence: 6299.4

Scaled residuals:

Min 1Q Median 3Q Max

-3.2618 -0.3936 0.2004 0.6690 2.2021

Random effects:

Groups Name Variance Std.Dev.

Aq_ID (Intercept) 32.51 5.701

Residual 137.07 11.708

Number of obs: 810, groups: Aq_ID, 15

Fixed effects:

Estimate Std. Error df t value Pr(>|t|)

(Intercept) 55.42923 3.49978 10.99459 15.838 6.46e-09 ***

treatment1 6.02129 4.83345 9.99989 1.246 0.241

treatment10 5.46278 4.83345 9.99989 1.130 0.285

treatment100 3.99496 4.83345 9.99989 0.827 0.428

treatment1000 2.90320 4.83345 9.99989 0.601 0.561

time -0.95643 0.07929 794.00004 -12.063 < 2e-16 ***

---

Signif. codes: 0 ‘***’ 0.001 ‘**’ 0.01 ‘*’ 0.05 ‘.’ 0.1 ‘ ’ 1

Correlation of Fixed Effects:

(Intr) trtmn1 trtm10 trt100 tr1000

treatment1 -0.691

treatment10 -0.691 0.500

treatmnt100 -0.691 0.500 0.500

tretmnt1000 -0.691 0.500 0.500 0.500

time -0.215 0.000 0.000 0.000 0.000

Type III Analysis of Variance Table with Satterthwaite's method

Sum Sq Mean Sq NumDF DenDF F value Pr(>F)

treatment 268.8 67.2 4 10 0.4902 0.7433

time 19944.1 19944.1 1 794 145.5063 <2e-16 ***

---

Signif. codes: 0 ‘***’ 0.001 ‘**’ 0.01 ‘*’ 0.05 ‘.’ 0.1 ‘ ’ 1

Simultaneous Tests for General Linear Hypotheses

Multiple Comparisons of Means: Dunnett Contrasts

Fit: lmer(formula = cummulative.no.movement.s ~ treatment + time +

(1 | Aq_ID), data = JBF.CIT.2_20)

Linear Hypotheses:

Estimate Std. Error z value Pr(>|z|)

1 - 0 == 0 6.021 4.833 1.246 0.535

10 - 0 == 0 5.463 4.833 1.130 0.618

100 - 0 == 0 3.995 4.833 0.827 0.825

1000 - 0 == 0 2.903 4.833 0.601 0.934

(Adjusted p values reported -- single-step method)

Groups Name Variance

Aq_ID (Intercept) 32.505

Residual 137.067

### Cortisol content juvenile brown trout

Linear mixed model fit by REML. t-tests use Satterthwaite's method [

lmerModLmerTest]

Formula: Cortisolgehalt ~ Treatment + Im.Verhaltensversuch. + (1 | Block)

Data: Data.CortisolCIT

REML criterion at convergence: 784.2

Scaled residuals:

Min 1Q Median 3Q Max

-1.5635 -0.6657 -0.1612 0.5083 3.0642

Random effects:

Groups Name Variance Std.Dev.

Block (Intercept) 19.72 4.441

Residual 153.06 12.372

Number of obs: 103, groups: Block, 3

Fixed effects:

Estimate Std. Error df t value Pr(>|t|)

(Intercept) 26.979 4.294 5.550 6.283 0.00102 **

Treatment1 -5.830 3.951 96.997 -1.476 0.14331

Treatment10 -8.849 3.951 96.997 -2.240 0.02741 *

Treatment100 -0.780 4.008 96.995 -0.195 0.84609

Treatment1000 6.883 3.970 87.715 1.734 0.08648 .

Im.Verhaltensversuch.nein -11.168 2.732 95.378 -4.088 9.07e-05 ***

---

Signif. codes: 0 ‘***’ 0.001 ‘**’ 0.01 ‘*’ 0.05 ‘.’ 0.1 ‘ ’ 1

Correlation of Fixed Effects:

(Intr) Trtmn1 Trtm10 Trt100 Tr1000

Treatment1 -0.428

Treatment10 -0.428 0.510

Treatmnt100 -0.427 0.504 0.504

Tretmnt1000 -0.470 0.397 0.397 0.390

Im.Vrhltns. -0.493 0.023 0.023 0.034 0.046

Type III Analysis of Variance Table with Satterthwaite's method

Sum Sq Mean Sq NumDF DenDF F value Pr(>F)

Treatment 2303.6 575.9 4 68.638 3.7625 0.007959 **

Im.Verhaltensversuch. 2558.2 2558.2 1 95.378 16.7132 9.072e-05 ***

---

Signif. codes: 0 ‘***’ 0.001 ‘**’ 0.01 ‘*’ 0.05 ‘.’ 0.1 ‘ ’ 1

Simultaneous Tests for General Linear Hypotheses

Multiple Comparisons of Means: Dunnett Contrasts

Fit: lmer(formula = Cortisolgehalt ~ Treatment + Im.Verhaltensversuch. +

(1 | Block), data = Data.CortisolCIT)

Linear Hypotheses:

Estimate Std. Error z value Pr(>|z|)

1 - 0 == 0 -5.830 3.951 -1.476 0.3939

10 - 0 == 0 -8.849 3.951 -2.240 0.0853 .

100 - 0 == 0 -0.780 4.008 -0.195 0.9991

1000 - 0 == 0 6.883 3.970 1.734 0.2521

---

Signif. codes: 0 ‘***’ 0.001 ‘**’ 0.01 ‘*’ 0.05 ‘.’ 0.1 ‘ ’ 1

(Adjusted p values reported -- single-step method)

# Number of fish

Table 10: number of individual fish assessed per parameter in the experiment with brown trout larvae exposed to citalopram. n.a. = not assessed

| parameter | brown trout larvae 7 °C | | | | | brown trout larvae 11 °C | | | | |
| --- | --- | --- | --- | --- | --- | --- | --- | --- | --- | --- |
| treatment | control | 1 µg/L | 10 µg/L | 100 µg/L | 1000 µg/L | control | 1 µg/L | 10 µg/L | 100 µg/L | 1000 µg/L |
| weight | 58 | 56 | 56 | 53 | 54 | 31 | 34 | 28 | 40 | 36 |
| lenght | 58 | 56 | 56 | 53 | 54 | 31 | 34 | 28 | 40 | 36 |
| heart rate | 10 | 15 | 15 | 15 | 15 | 15 | n.a. | n.a. | n.a. | 15 |
| mortality | 90 | 90 | 90 | 90 | 90 | 90 | 90 | 90 | 90 | 90 |
| time to hatch | 90 | 90 | 90 | 87 | 89 | 90 | 89 | 90 | 90 | 90 |
| Behavior during exposure | 30 fish * 2 tanks * 12 days | 30 fish * 1 tanks * 12 days | 30 fish * 2 tanks * 12 days | 30 fish * 1 tanks * 12 days | 30 fish * 2 tanks * 12 days | 20 fish * 2 tanks * 8 days | n.a. | 20 fish * 2 tanks * 8 days | 20 fish * 2 tanks * 8 days | 20 fish * 2 tanks * 8 days |
| ASMD | 15 | 15 | 15 | 15 | 15 | 15 | 15 | 15 | 15 | 15 |
| Tissue citalopram concentration | 10 individuals per block pooled | | | | | 10 individuals per block pooled | | | | |

Table 11: number of individual fish assessed per parameter in the experiment with juvenile brown trout exposed to citalopram.

| parameter | juvenile brown trout | | | | |
| --- | --- | --- | --- | --- | --- |
| treatment | control | 1 µg/L | 10 µg/L | 100 µg/L | 1000 µg/L |
| weight | 30 | 30 | 30 | 30 | 30 |
| lenght | 30 | 30 | 30 | 30 | 30 |
| mortality | 30 | 30 | 30 | 30 | 30 |
| Behavior during exposure | 10 fish * 2 tanks * 12 days | 10 fish * 2 tanks * 12 days | 10 fish * 1 tanks * 12 days | 10 fish * 2 tank * 12 days | 10 fish * 2 tanks * 12 days |
| ASMD: total distance moved | 9 | 9 | 9 | 9 | 9 |
| ASMD: mean velocity | 9 | 9 | 9 | 9 | 9 |
| Cortisol content | 21 | 20 | 20 | 19 | 23 |
| Cortisol content between fish tested and not tested in the ASMD | | tested: 29 | | not tested: 74 | |
| Tissue citalopram concentration | 10 individuals per block pooled | | | | |
