# Supplementary material for: Impact of the antidepressant citalopram on the behaviour of two different life stages of brown trout
Source: PeerJ. 2020 Mar 12;8:e8765. doi: 10.7717/peerj.8765 (PMC7073243; doi:10.7717/peerj.8765)
Supplement: Supplemental Information 4 [file peerj-08-8765-s004.docx]

# Water conditions

Table 4: Water quality parameters during the experiment with brown trout larvae exposed to citalopram. mean values are shown.

| sampling-date | climate chamber | treatment citalopram | oxygen cocentration [mg/L] | oxygen cocentration [%] | pH | conductivity [µs/cm] | temperature [°C] | Ammonium level [NH_4_^+^ mg/L] |
| --- | --- | --- | --- | --- | --- | --- | --- | --- |
| 28.12.2016 | 7 °C | control | 11.21 | 95.60 | 7.22 | 466.33 | 7.27 | - |
| 28.12.2016 | 7 °C | 1 µg/L | 11.09 | 94.93 | 7.81 | 465.33 | 7.43 | - |
| 28.12.2016 | 7 °C | 10 µg/L | 11.24 | 95.30 | 7.98 | 462.33 | 7.07 | - |
| 28.12.2016 | 7 °C | 100 µg/L | 11.07 | 94.77 | 7.98 | 462.67 | 7.47 | - |
| 28.12.2016 | 7 °C | 1000 µg/L | 11.18 | 94.97 | 8.01 | 459.33 | 7.13 | - |
| 28.12.2016 | 11 °C | control | 10.48 | 96.03 | 7.47 | 473.33 | 10.27 | - |
| 28.12.2016 | 11 °C | 1 µg/L | 10.36 | 95.47 | 7.59 | 470.33 | 10.50 | - |
| 28.12.2016 | 11 °C | 10 µg/L | 10.46 | 95.77 | 7.89 | 467.33 | 10.23 | - |
| 28.12.2016 | 11 °C | 100 µg/L | 10.36 | 95.57 | 7.86 | 470.33 | 10.57 | - |
| 28.12.2016 | 11 °C | 1000 µg/L | 10.43 | 95.70 | 7.92 | 466.67 | 10.37 | - |
| 18.01.2017 | 7 °C | control | 10.73 | 93.13 | 7.16 | 467.67 | 7.03 | - |
| 18.01.2017 | 7 °C | 1 µg/L | 10.68 | 92.90 | 7.96 | 467.00 | 7.27 | - |
| 18.01.2017 | 7 °C | 10 µg/L | 10.82 | 93.20 | 8.05 | 464.67 | 7.03 | - |
| 18.01.2017 | 7 °C | 100 µg/L | 10.69 | 92.80 | 8.02 | 466.67 | 7.23 | - |
| 18.01.2017 | 7 °C | 1000 µg/L | 10.84 | 93.00 | 8.13 | 465.33 | 6.90 | - |
| 18.01.2017 | 11 °C | control | 10.12 | 93.63 | 7.44 | 476.00 | 10.13 | - |
| 18.01.2017 | 11 °C | 1 µg/L | 10.05 | 93.43 | 7.60 | 474.33 | 10.30 | - |
| 18.01.2017 | 11 °C | 10 µg/L | 10.12 | 93.47 | 7.95 | 472.00 | 10.10 | - |
| 18.01.2017 | 11 °C | 100 µg/L | 10.00 | 93.17 | 7.93 | 474.67 | 10.43 | - |
| 18.01.2017 | 11 °C | 1000 µg/L | 10.04 | 93.33 | 8.04 | 472.00 | 10.43 | - |
| 06.03.2017 | 7 °C | control | 10.53 | 93.80 | 7.19 | 473.33 | 7.03 | - |
| 06.03.2017 | 7 °C | 1 µg/L | 10.24 | 92.50 | 7.80 | 473.67 | 7.40 | - |
| 06.03.2017 | 7 °C | 10 µg/L | 10.32 | 92.33 | 7.89 | 471.00 | 7.07 | - |
| 06.03.2017 | 7 °C | 100 µg/L | 10.36 | 93.10 | 7.97 | 472.67 | 7.20 | - |
| 06.03.2017 | 7 °C | 1000 µg/L | 10.50 | 93.67 | 8.02 | 472.67 | 7.03 | - |
| 06.03.2017 | 11 °C | control | 9.74 | 93.93 | 7.39 | 485.67 | 10.43 | - |
| 06.03.2017 | 11 °C | 1 µg/L | 9.68 | 93.63 | 7.56 | 484.33 | 10.50 | - |
| 06.03.2017 | 11 °C | 10 µg/L | 9.66 | 93.20 | 7.88 | 481.67 | 10.37 | - |
| 06.03.2017 | 11 °C | 100 µg/L | 9.59 | 93.13 | 7.89 | 485.00 | 10.57 | - |
| 06.03.2017 | 11 °C | 1000 µg/L | 9.75 | 93.83 | 7.99 | 483.33 | 10.57 | - |
| 13.04.2017 | 11 °C | control | 9.76 | 93.30 | 7.92 | 485.67 | 10.90 | 1,36 |
| 13.04.2017 | 11 °C | 1 µg/L | 9.81 | 93.17 | 7.89 | 486.33 | 10.63 | 1,5 |
| 13.04.2017 | 11 °C | 10 µg/L | 8.85 | 83.73 | 7.86 | 483.00 | 10.53 | 1,27 |
| 13.04.2017 | 11 °C | 100 µg/L | 9.77 | 93.17 | 7.82 | 486.33 | 10.80 | 1,44 |
| 13.04.2017 | 11 °C | 1000 µg/L | 9.79 | 93.03 | 7.78 | 487.33 | 10.70 | 0,86 |
| 12.05.2017 | 7 °C | control | 10.83 | 95.00 | 8.41 | 489.67 | 6.93 | 1,3 |
| 12.05.2017 | 7 °C | 1 µg/L | 10.76 | 94.67 | 8.48 | 490.33 | 6.97 | 1,4 |
| 12.05.2017 | 7 °C | 10 µg/L | 10.79 | 94.63 | 8.50 | 487.00 | 6.80 | 1,45 |
| 12.05.2017 | 7 °C | 100 µg/L | 10.78 | 94.70 | 8.50 | 491.00 | 6.90 | 1,35 |
| 12.05.2017 | 7 °C | 1000 µg/L | 10.77 | 94.30 | 8.49 | 483.33 | 6.87 | 1,32 |

Table 5: Water quality parameters at the beginning and the end of the experiment with juvenile brown trout exposed to citalopram. mean values are shown.

| sampling-date | treatment citalopram | oxygen concentration [mg/L] | oxygen concentration [%] | pH | conductivity [µS/cm] | temperature [°C] | Ammonium level [NH_4_^+^ mg/L] |
| --- | --- | --- | --- | --- | --- | --- | --- |
| 08.08.2017 | control | 11.16 | 97.47 | 8.10 | 476.00 | 7.30 | - |
| 08.08.2017 | 1 µg/L | 11.15 | 97.70 | 8.10 | 478.67 | 7.37 | - |
| 08.08.2017 | 10 µg/L | 11.28 | 97.97 | 8.09 | 474.00 | 7.00 | - |
| 08.08.2017 | 100 µg/L | 11.29 | 97.80 | 8.08 | 478.67 | 6.90 | - |
| 08.08.2017 | 1000 µg/L | 11.22 | 97.90 | 8.07 | 474.67 | 7.20 | - |
| 04.09.2017 | control | 11.29 | 98.27 | 8.13 | 511.67 | 7.07 | >3 |
| 04.09.2017 | 1 µg/L | 11.32 | 98.50 | 8.14 | 513.00 | 7.20 | >3 |
| 04.09.2017 | 10 µg/L | 11.35 | 98.00 | 8.13 | 509.67 | 6.90 | >3 |
| 04.09.2017 | 100 µg/L | 11.38 | 98.07 | 8.13 | 511.33 | 6.80 | >3 |
| 04.09.2017 | 1000 µg/L | 11.35 | 98.10 | 8.14 | 509.67 | 6.93 | >3 |

Water concentration

Table 6: citalopram water concentration (µg/l) in the test aquaria of the experiment with brown trout larvae exposed to citalopram, measured with LC-MS. mean values are shown. b.w.e. = before water exchange. a.w.e. = after water exchange. n.m. = not measured. LOD = limit of detection

| climate chamber | treatment | 28.12.2016 | 20.01.2017 b.w.e. | 20.01.2017 a.w.e. | 14.02.2017 b.w.e. | 14.02.2017 a.w.e. | 07.03.2017 b.w.e. |
| --- | --- | --- | --- | --- | --- | --- | --- |
| 7°C | control | <LOD | <LOD | <LOD | <LOD | <LOD | <LOD |
| 7°C | 1 µg/L | 0.84 | 0.56 | 0.69 | 0.91 | 1.63 | 0.72 |
| 7°C | 10 µg/L | 7.96 | 8.32 | 8.27 | 8.33 | 9.26 | 9.44 |
| 7°C | 100 µg/L | 71.85 | 92.83 | 82.09 | 61.75 | 60.09 | 52.04 |
| 7°C | 1000 µg/L | 939.00 | 960.00 | 1097.00 | 941.38 | 848.74 | 928.19 |
| 7°C | degradation control  100 µg/L | 82.57 | 80.43 | 76.52 | n.m. | n.m. | n.m. |
| 11°C | control | <LOD | <LOD | <LOD | <LOD | <LOD | <LOD |
| 11°C | 1 µg/L | 0.69 | 0.52 | 0.71 | 0.76 | 0.84 | 0.71 |
| 11°C | 10 µg/L | 7.85 | 5.89 | 7.09 | 8.47 | 9.20 | 7.69 |
| 11°C | 100 µg/L | 71.32 | 66.22 | 61.27 | 71.06 | 63.20 | 55.25 |
| 11°C | 1000 µg/L | 802.00 | 717.00 | 1185.00 | 883.15 | 909.49 | 804.16 |
| climate chamber | treatment | 07.03.17 b.w.e. | 07.03.17 a.w.e. | 04.04.17 b.w.e. | 04.04.17 a.w.e. | 13.04.2017 | 12.05.2017 |
| 7°C | control | <LOD | <LOD | <LOD | <LOD | <LOD | <LOD |
| 7°C | 1 µg/L | 0.72 | 0.85 | 0.68 | 0.76 | 0.77 | 0.73 |
| 7°C | 10 µg/L | 9.44 | 8.36 | 8.91 | 9.20 | 9.18 | 8.93 |
| 7°C | 100 µg/L | 52.04 | 62.22 | 80.26 | 73.10 | 73.13 | 66.18 |
| 7°C | 1000 µg/L | 928.19 | 852.83 | 1105.50 | 1242.00 | 1150.51 | 1132.48 |
| 11°C | control | <LOD | <LOD | <LOD | <LOD | <LOD | n.m. |
| 11°C | 1 µg/L | 0.71 | 0.92 | 1.30 | 0.73 | 0.75 | n.m. |
| 11°C | 10 µg/L | 7.69 | 8.48 | 10.30 | 8.93 | 9.06 | n.m. |
| 11°C | 100 µg/L | 55.25 | 58.96 | 74.39 | 66.10 | 69.65 | n.m. |
| 11°C | 1000 µg/L | 804.16 | 894.35 | 1219.00 | 1223.00 | 1102.60 | n.m. |

Table 7: citalopram water concentration (µg/L) in the testaquaria of the experiment with juvenile brown trout exposed to citalopram. measured with LC-MS. mean values are shown. b.w.e. = before water exchange. a.w.e. = after water exchange. LOD = Limit of detection

| treatment | 08.08.2017 | 21.08.2017 b.w.e. | 21.08.2017 a.w.e. | 04.09.2017 |
| --- | --- | --- | --- | --- |
| control | <LOD | <LOD | <LOD | <LOD |
| 1 µg/L | 1.12 | 1.67 | 1.26 | 1.58 |
| 10 µg/L | 8.77 | 9.50 | 10.02 | 8.51 |
| 100 µg/L | 82.80 | 84.41 | 80.84 | 77.99 |
| 1000 µg/L | 865.29 | 885.52 | 925.09 | 783.81 |

Table 8: calculation of plasma concentration according to Schreiber et al. (2011) at specific pH 8

| life stage | temperature | treatment | plasma concentration (µg/L) (calculated according to Schreiber et al. (2011)) |
| --- | --- | --- | --- |
| brown trout larvae | 11°C | 1 µg/L | 2.91 |
| brown trout larvae | 11°C | 10 µg/L | 24.88 |
| brown trout larvae | 11°C | 100 µg/L | 197.09 |
| brown trout larvae | 11°C | 1000 µg/L | 2920.00 |
| brown trout larvae | 7°C | 1 µg/L | 2.49 |
| brown trout larvae | 7°C | 10 µg/L | 26.20 |
| brown trout larvae | 7°C | 100 µg/L | 211.36 |
| brown trout larvae | 7°C | 1000 µg/L | 3051.67 |
| juvenile brown trout | 7°C | 1 µg/L | 4.23 |
| juvenile brown trout | 7°C | 10 µg/L | 27.58 |
| juvenile brown trout | 7°C | 100 µg/L | 244.37 |
| juvenile brown trout | 7°C | 1000 µg/L | 2593.07 |

**
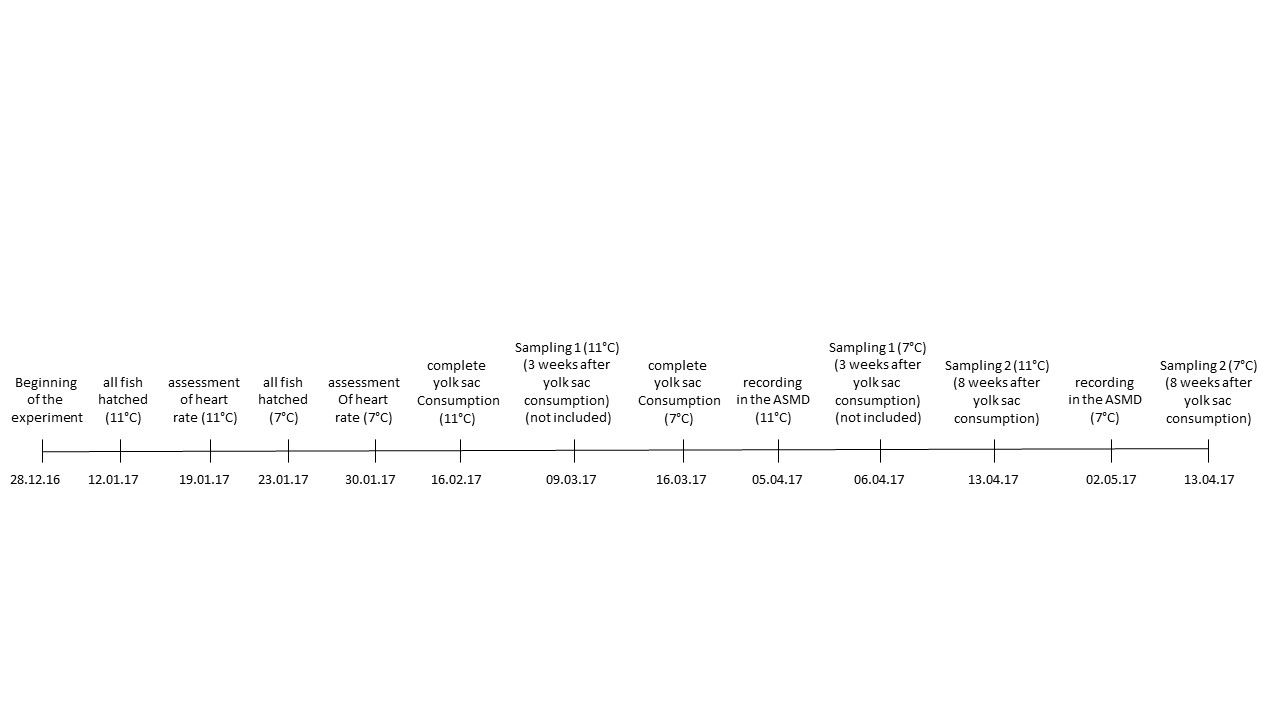
Timeline experiment with brown trout larvae**
